# Supplementary material for: Ilimaquinone Induces the Apoptotic Cell Death of Cancer Cells by Reducing Pyruvate Dehydrogenase Kinase 1 Activity
Source: Int J Mol Sci. 2020 Aug 21;21(17):6021. doi: 10.3390/ijms21176021 (PMC7504051; doi:10.3390/ijms21176021)
Supplement: Supplementary file 1 [file ijms-21-06021-s001.pdf]

International Journal of *Molecular Sciences*

MDPI

Supplementary Materials

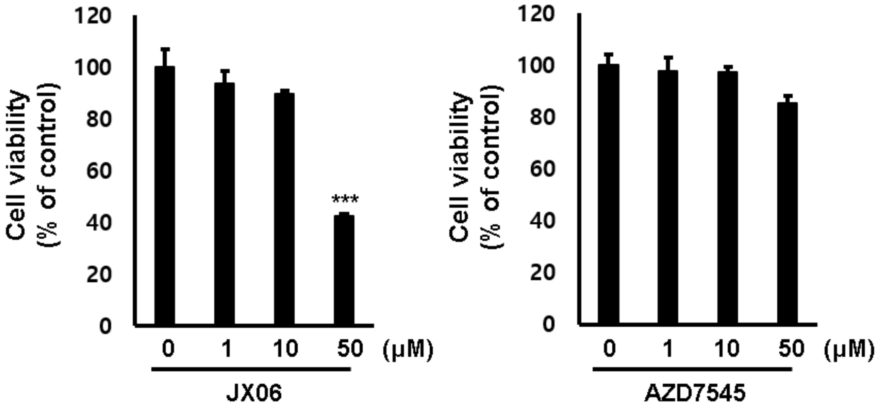

Supplementary Figure S1. The cell viabilities of JX06, and AZD7545 in A549 cells. The cells were treated with JX06, and AZD7545 for 24 h. All experiments were performed at least three times, and data shown as the mean ± SD. Statistical analyses were performed using Student's *t*-test. ***, $p < 0.001$ compared with the control.

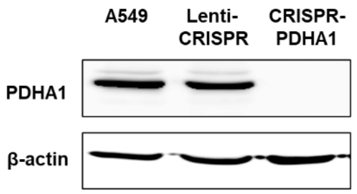

Supplementary Figure S2. Construction of PDHA1-knockout A549 cells. The A549 cells were infected with lentivirus harboring mock CRISPR vector or PDHA1-knockout vector. The protein expression levels of PDHA1 were determined via western blotting.

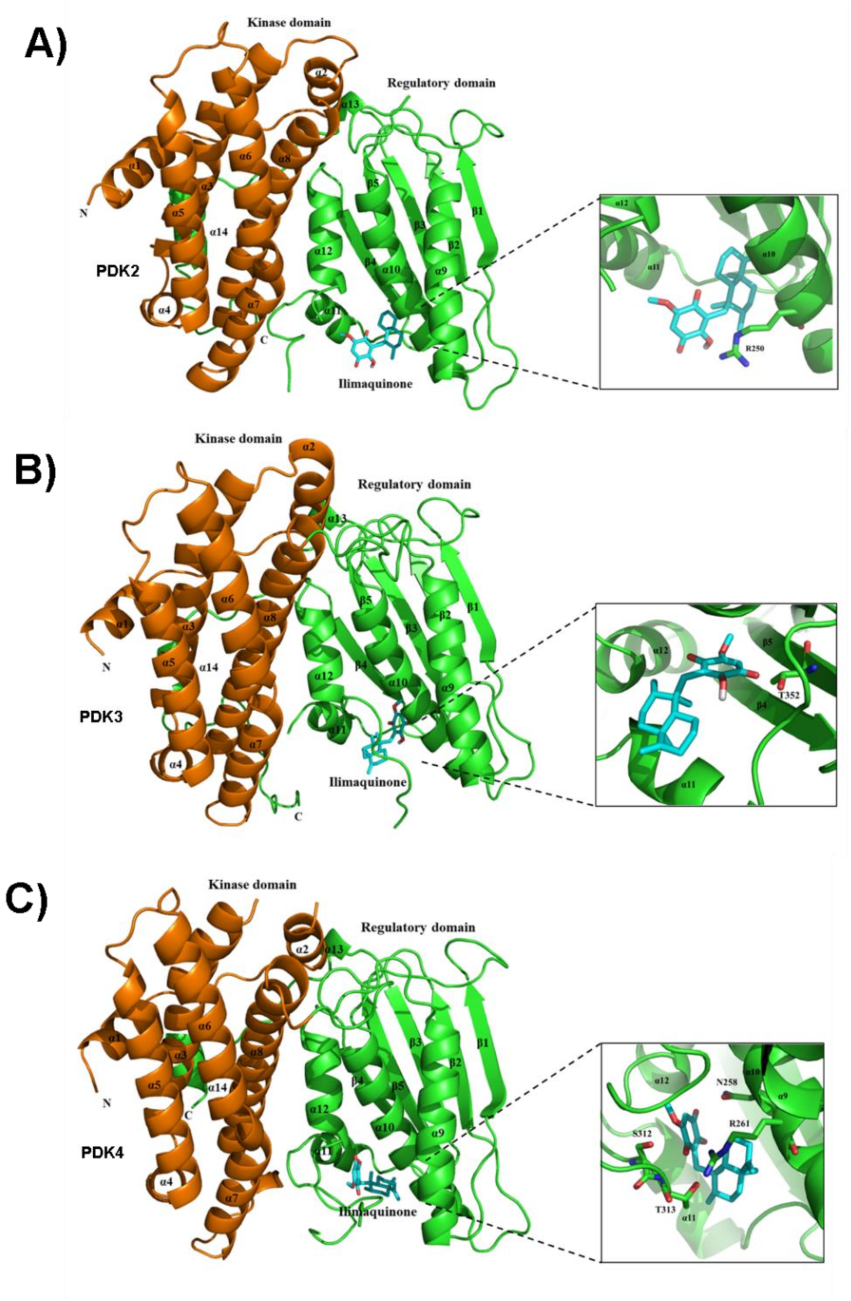

D)

PDK1 MVFELFKNAMRATMEHHANRG-VYPPIQVHVTLGNEDLTVKMSDRGGGVPLRKIDRLFNYMYSTAPRPRVETSRAVPLAGFGYGLP

PDK2 MLFELFKNAMRATVESHESSL-ILPPIKVMVALGEEDLSIKMSDRGGGVPLRKIERLFSYMYSTAPTPQPGTG-GTPLAGFGYGLP

PDK3 MLFELFKNSMRATVELYEDRKEGYPAVKTLVTLGKEDLSIKISDLGGGVPLRKIDRLFNYMYSTAPRPSLEPTRAAPLAGFGYGLP

PDK4 MLFELFKNAMRATVEHQENQP-SLTPIEVIVVLGKEDLTIKISDRGGGVPLRIIDRLFSYTYSTAPTPVMDNSRNAPLAGFGYGLP

ATP binding site
IQ binding residue (predicted)

Supplementary Figure S3. The interaction of PDK isozymes with IQ. The modeled structures of PDK isoforms 2 (PDB ID: 2BTZ), 3 (PDB ID:1Y8N), and 4 (PDB ID: 2E0A) with IQ (CID: 21727418) are shown as ribbons. The interaction residues between a PDK isoform and IQ are shown.

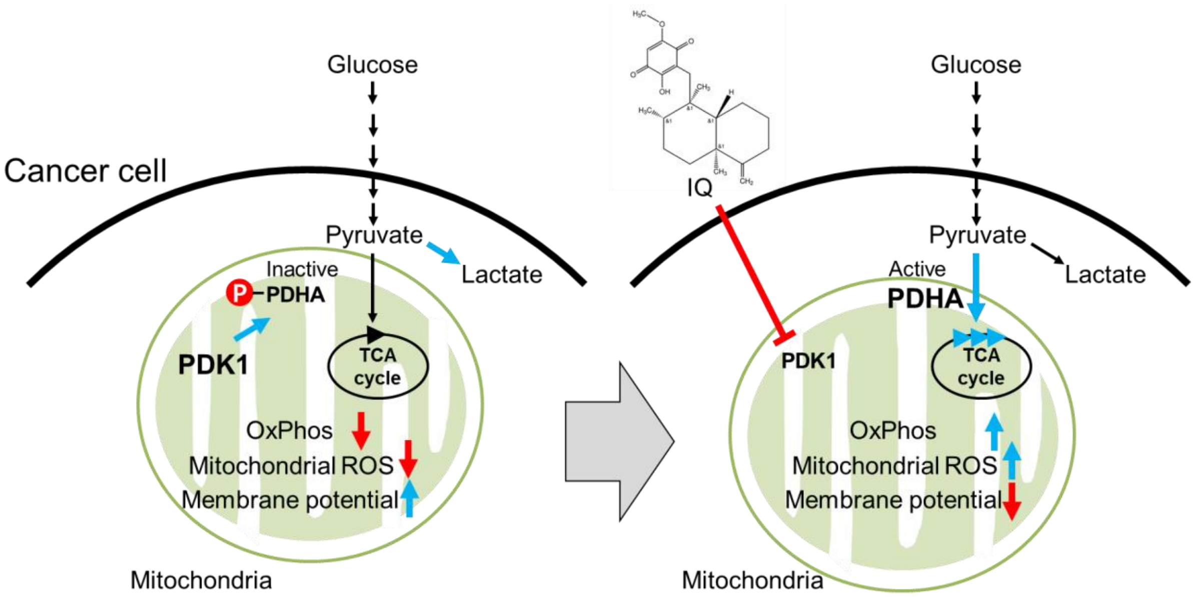

Supplementary Figure S4. Schematic diagram of the mechanism of IQ-mediated apoptosis. The red circle and white P indicate phosphates.
